# Digital interventions addressing the unmet needs of older adults with multimorbidity: a mixed-methods persona design approach

**DOI:** 10.3389/fpubh.2025.1637748

**Published:** 2025-11-17

**Authors:** Vincenzo De Luca, Michele Virgolesi, Alessandra Cuomo, Daniela Lemmo, Mariangela Perillo, Federica Canfora, Sara Aprano, Fabrizio Mezza, Claudia Vetrani, Maria Francesca Freda, Daniela Adamo, Teresa Rea, Lorenzo Mercurio, Erminia Attaianese, Enrica Menditto, Annamaria Colao, Guido Iaccarino, Maddalena Illario

**Affiliations:** 1 Dipartimento di Sanità Pubblica, Università degli Studi di Napoli Federico II, Napoli, Italy; 2 Dipartimento di Medicina Clinica e Chirurgia, Università degli Studi di Napoli Federico II, Napoli, Italy; 3 Dipartimento di Studi Umanistici, Università degli Studi di Napoli Federico II, Napoli, Italy; 4 Dipartimento di Architettura, Università degli Studi di Napoli Federico II, Napoli, Italy; 5 Dipartimento di Neuroscienze e Scienze Riproduttive ed Odontostomatologiche, Università degli Studi di Napoli Federico II, Napoli, Italy; 6 Centro Italiano per la Cura e il Benessere del Paziente con Obesità (C.I.B.O), Azienda Ospedaliera Universitaria Federico II, Napoli, Italy; 7 Dipartimento di Benessere, Nutrizione e Sport, Università Telematica Pegaso, Napoli, Italy; 8 Dipartimento di Farmacia, Università degli Studi di Napoli Federico II, Napoli, Italy

**Keywords:** older adults, multimorbidity, frailty, polypharmacy, digital health, telemedicine, persona-based design, mixed-methods

## Abstract

**Background:**

Frailty and multimorbidity in older adults require integrated, multidomain care models. Digital health solutions offer potential for improving self-management, but a structured methodology to translate the complex unmet needs of this population into tailored digital interventions is still lacking.

**Objectives:**

To propose and illustrate a mixed-methods, persona-based approach for mapping unmet needs of older adults with multimorbidity to candidate digital interventions.

**Methods:**

This study employed an explanatory sequential mixed-methods design. First, quantitative data from the SUNFRAIL+ frailty screening tool were analyzed for a sub-cohort of community-dwelling older adults with polypharmacy (*n* = 54). These findings informed a qualitative phase conducted through a multidisciplinary Focus Group (FG) of 15 healthcare and social care experts. FG transcripts were thematically analyzed to identify unmet needs across clinical, psychological, social, and environmental domains. These insights were synthesized using the European Commission’s Blueprint methodology to create a realistic persona, “Gennaro,” representing common challenges. Finally, the FG systematically mapped potential digital solutions to address the identified needs. We descriptively profiled a polypharmacy subgroup (*n* = 54) from the F2UH site of the multicentre SUNFRAIL+ study; no hypothesis testing was performed.

**Results:**

SUNFRAIL+ data revealed a high prevalence of perceived memory loss (44.4%), reduced physical activity (40.7%), loneliness (29.6%), and gaps in primary care access (25.9%). Thematic analysis highlighted unmet needs in medication management, motivation for lifestyle change, social isolation, and environmental barriers. The persona “Gennaro” embodied these challenges, facilitating the identification of targeted digital solutions, including Shared Care Plans, Telemonitoring, smart pillboxes for Medication Adherence, Adapted Physical Activity programs, Motivational Interviewing apps, and Ambient Assisted Living (AAL) systems. A foundational dataset and interoperability requirements for implementation were also defined.

**Conclusion:**

This descriptive, hypothesis-generating study integrates quantitative screening and multidisciplinary expert insights to propose a structured, persona-guided approach for profiling the multifaceted unmet needs of older adults with multimorbidity. We outline candidate digital options mapped to specific challenges as a preliminary framework to hypothesize person-centered, digitally supported care models. End-user co-design and validation are planned next to evaluate feasibility, usability, acceptability, and potential impact on self-management and adherence.

## 1 Introduction

Multimorbidity, defined as the coexistence of more than one chronic condition in a single individual (1), is increasingly common in older adults, with prevalence >75% after 65 years (2). Its prevalence increases with age, exhibits gender disparities (3), and is a global phenomenon observed in both high- and low-income countries (4). Frailty, an age-related multifactorial condition, increases the risk of hospitalization, disability and death (5). It frequently coexists with multimorbidity (6) and contributes to reduced quality of life and mortality mainly from cardiovascular, oncological, respiratory and metabolic diseases.

Older adults with multimorbidity, especially when frail, show frequent hospitalizations and require complex, structured care plans that address overall health beyond single-disease management (7–9). Therapeutic adherence is a key determinant in managing multimorbidity and polypharmacy: low adherence worsens quality of life and increases costs, while effective interventions must be tailored to individual and contextual risk factors (10–12).

Given that frailty and multimorbidity affect nearly half of the older population (46.2%), early diagnosis, prevention, and integrated management are healthcare priorities to sustain healthcare systems and improve self-management (13). Managing multiple chronic diseases requires sustained psychological adaptation and coping skills (14–16). Social relationships and socioeconomic conditions are equally crucial, as disadvantage and barriers in access to services increase health disparities and vulnerability in older adults (17–20). Addressing multimorbidity requires holistic, integrated strategies across health and social services, supported by data sharing and personalized care plans (21–24). Digital health can facilitate self-management, adherence, and communication with professionals, while also supporting diagnosis, therapy, and clinical decision-making (25, 26). Its effectiveness, however, depends on health literacy, usability, and contextual adaptation, as adoption is limited by organizational, technical, economic, and cultural barriers (27–31). In this context, the European Commission’s ‘Blueprint on the Digital Transformation of Health and Care for an Ageing Society’ provides a structured methodology to define demand-driven use cases and personas that capture unmet needs, possible IT solutions, and enabling technologies for active and healthy ageing (32, 33). This study aimed to identify the socio-health unmet needs of community-dwelling older adults with multimorbidity and polypharmacy using a mixed-methods approach. A secondary objective was to translate these findings into a structured Blueprint persona and systematically map potential digital health solutions to address the identified needs.

## 2 Materials and methods

The study implemented a mixed qualitative-quantitative method aimed at identifying and analyzing key digital solutions and high-impact usage scenarios applied to community-dwelling older adults with two or more chronic conditions. A Focus Group (FG) was set up at Federico II University and Hospital (F2UH) to empathetically engage key professionals with extensive knowledge of healthcare services for older patients with multimorbidity, aimed at identifying care processes that could be enhanced by digital tools (34, 35). This was a mixed-methods study that combined quantitative screening data from the SUNFRAIL+ tool with qualitative insights from a multidisciplinary Focus Group. The quantitative component provided a profile of risk factors, while the qualitative component enabled an in-depth exploration of unmet needs, which were subsequently translated into digital health requirements.

### 2.1 Focus group—procedure

A multidisciplinary professionals’ focus group was conducted at F2UH with participants from internal medicine (*n* = 2), endocrinology (*n* = 1), geriatrics (*n* = 1), nursing (*n* = 1), pharmacy (*n* = 1), psychology (*n* = 1), nutrition (*n* = 3), biology (*n* = 1), digital health/biomedical engineering (*n* = 2), and architecture (*n* = 2). Professionals were recruited via purposive sampling (role/seniority and direct involvement in the care of older adults with multimorbidity at F2UH); the sample size was determined pragmatically to ensure multidisciplinary coverage and was deemed adequate by thematic sufficiency across four sessions. The FG met four times (Sept 2023–Mar 2024) and followed a semi-structured guide covering: (i) unmet needs relevant to multimorbidity, (ii) candidate digital functions and data/interoperability requirements, and (iii) barriers/facilitators and acceptability. Each session lasted approximately 90 min, was audio-recorded, and a rapporteur kept structured minutes on a standardized electronic template; outputs were member-checked by participants before consolidation. The verbatim prompts used with professionals and the stimulus technologies are provided in Supplementary material S1. Any disagreements on item wording or categorization were noted in the minutes and finalized in a subsequent consensus meeting; unresolved items were adjudicated by a third senior investigator.

### 2.2 Qualitative analysis

To analyze the focus-group discussions, audio recordings were transcribed verbatim and examined using reflexive thematic analysis following Braun & Clarke’s phases: (i) familiarization; (ii) generation of initial codes; (iii) construction of candidate themes; (iv) review of themes against coded extracts and the full dataset; (v) theme definition and naming; and (vi) reporting. Two researchers independently coded all materials using an inductive, primarily semantic approach (allowing latent insights when warranted). A shared codebook was iteratively developed and refined; discrepancies were resolved by consensus, with escalation to a third senior investigator when needed. To support trustworthiness, we maintained an audit trail (audio files, structured minutes, versioned matrices), sought negative/contradictory cases, and conducted a member-check of key takeaways at the end of sessions. In line with a reflexive approach to thematic analysis, we did not compute inter-rater reliability coefficients (e.g., Cohen’s *κ*); rigor was ensured through double coding, consensus meetings, and transparent documentation. The codebook (labels and definitions) and a textual theme map are provided in Supplementary material S2; anonymized exemplar extracts can be supplied to editors upon reasonable request.

### 2.3 SUNFRAIL+

In this manuscript, we analysed SUNFRAIL+ screening data collected at the Federico II University Hospital (F2UH) site. Patients with multiple chronic conditions are more likely to receive multiple drug treatments (36); accordingly, many studies use two or more conditions (multimorbidity) and five or more medications (polypharmacy) as consistent endpoints (37). The Focus Group (FG) reviewed preliminary data from the multicentre SUNFRAIL+ study promoted by the PROMIS network (38), with contributions from eight Italian regions and coordinated by F2UH (39). The study aimed to validate an innovative model of frailty screening in community-dwelling older adults, supported by information technology (IT). The screening model adopts the SUNFRAIL tool, consisting of nine questions investigating the physical, psychological, and socio-economic domains (Table 1) (40). After written informed consent, screening data were collected by clinicians during outpatient visits across five F2UH departments (Adapted Physical Activity Prescription, Geriatrics, Neurology, Diabetology, Maxillofacial Surgery). A total of *n* = 113 older adults were enrolled at the F2UH site of the multicentre SUNFRAIL+ study (see Ethical approval, Section 2.4). Analyses focused on the polypharmacy subgroup (*n* = 54) and were strictly descriptive. Given this descriptive aim, no *a priori* power calculation was performed. To profile common characteristics, the FG selected older adults who answered “yes” to the question “Do you regularly take five or more medications per day?” and identified the frailty risk factors they presented. Only counts and percentages are reported; no inferential statistics (e.g., *p*-values, confidence intervals) were computed.

**Table 1:** The SUNFRAIL checklist (40).

Questions	Answers	
1. Do you regularly take 5 or more medications per day?	Yes (Alert)	No
2. Have you recently lost weight such that your clothing has become looser?	Yes (Alert)	No
3. Your physical state made you walking less during the last year?	Yes (Alert)	No
4. Have you been evaluated by your GP during the last year?	Yes	No (Alert)
5. Have you fallen 1 or more times during the last year?	Yes (Alert)	No
6. Have you experienced memory decline during the last year?	Yes (Alert)	No
7. Do you feel lonely most of the time?	Yes (Alert)	No
8. In case of need, can you count on someone close to you?	Yes	No (Alert)
9. Have you had any financial difficulties in facing dental care and health care costs during the last year?	Yes (Alert)	No

### 2.4 Blueprint persona

The European Commission’s Blueprint methodology is a patient-centred approach for the design of IT-supported interventions that highlight patients’ unmet needs to design key use scenarios (41). The health, social and technological needs of the target population are represented through a “Persona” use case, a single, specific, hypothetical patient, with a realistic name and a brief description of their needs, goals, hopes, dreams and attitudes (42). In addition, the use case reports the typical behavioral characteristics of the target population, based on the experience of the experts contributing to the definition. Based on the analysis of the risk factors resulting from SUNFRAIL+ questionnaire, a theoretical elaboration of the Blueprint persona was developed by the FG. The professionals involved in the FG answered iteratively to the questions in the Blueprint Persona Development tool (Supplementary material S3) and agreed on the final response. Persona development followed the European Commission’s Blueprint methodology. The process combined SUNFRAIL+ data with qualitative insights from the FG to build a realistic profile, including socio-demographic characteristics, health status, behavioural traits, and contextual factors. The persona (‘Gennaro’) was iteratively refined until consensus was reached among FG members. This persona represents an expert-informed synthesis step intended to structure design hypotheses. End-user co-design and validation were not undertaken at this stage and are planned as a subsequent phase within the broader UCD (User-Centered Design) pipeline.

### 2.5 Design of digital intervention

Based on the Persona use case, the FG identified digital solutions that can help meet the needs of patients in healthcare and other domains, such as social, psychological, environmental, etc., to design a digitally supported intervention, integrated into the current service provision, from organizational and technological perspectives. Finally, the FG identified the dataset to be collected through digitally supported intervention. In particular, the FG focused on the type of data, the tools adopted for data collection, the setup and interoperability requirements. Such a minimum dataset helps to collect and manage data from multiple sources (EHR, medical devices, wearables, etc.), identifying the interoperability requirements that digitally supported interventions must provide.

### 2.6 Ethical approval

The studies involving humans were approved by the local Ethics Committee of University “Federico II”—Azienda Ospedaliera di Rilievo Nazionale “A. Cardarelli” (N. 284/22). The research protocol was registered at ClinicalTrials.gov on 9 December 2022 (registration number: NCT05646472). The studies were conducted in accordance with local legislation and institutional requirements. Written informed consent for participation was obtained from the participants’ legal guardians or next of kin.”

## 3 Results

### 3.1 SUNFRAIL+ questionnaire results

The FG extracted and analyzed preliminary data of the SUNFRAIL+ study collected at the F2UH, the answers provided by *n* = 54 patients who declared they took 5 or more drugs. These data were not intended to provide inferential statistics, but rather to generate a descriptive profile of multimorbid patients with polypharmacy, which served to contextualize and enrich the identification of unmet needs in the subsequent qualitative phase. The mean age of this subpopulation is 74.68 (±6.01) and an age range between 66 and 95. More than half of the participants (55.5%) are aged 74 years or younger. *n* = 33 are women. Half of participants (50%; *n* = 27) have a low level of education. Only *n* = 5 (9.2%) had attained a university degree (Table 2). The most recurrent risk factor in this sub-population is the perception of memory loss (*n* = 24; 44.4%). This finding corresponds to the cognitive domain of the SUNFRAIL+ tool, highlighting early vulnerability that may interact with pharmacological complexity. This symptom could be linked both to the age-related loss of cognitive function and to the side effects of polypharmacy. In 40.7% of cases, participants reported a decrease in physical activity and the adoption of a more sedentary lifestyle, linked to health conditions and loss of independence. This result is aligned with the physical activity domain of SUNFRAIL+, reinforcing the link between reduced mobility, frailty progression, and loss of independence. The feeling of loneliness found in *n* = 16 participants (29.6%) highlight a vulnerable psychological state and a weakening of social relationships due to the loss of independence. This indicator belongs to the social domain of SUNFRAIL+, underlining how psychological vulnerability and social isolation interact with health status. 25.9% (*n* = 14) of participants declared that they had not been visited by their General Practitioner (GP) in the last year. This could be explained by difficult communication with GP, or a problem with the accessibility of doctor’s outpatient clinic. Finally, it could be a question of low trust in GP and inappropriate use of specialist services (Table 3). Overall, these descriptive results provided an empirical foundation across the main SUNFRAIL+ domains (cognitive, physical, social, and healthcare access). They guided the Focus Group in selecting the most relevant unmet needs for persona development and digital health solution mapping.”

**Table 2:** Enrolled older adults distribution by age, sex and level of education.

Characteristic	Value
Age (years), mean (SD)	74.68 (±6.01)
Gender, female, n (%)	33 (61.1)
Minimum age in years	66
Maximum age in years	95
Age classes, *n* (%)	
≤74	30 (55.5)
75–84	20 (37.0)
≥85	4 (7.4)
Level of education, *n* (%)	
Low (without studies, Primary school)	27 (50.0)
Medium (secondary school)	22 (40.7)
High (University, Master or PhD degree)	5 (9.2)

**Table 3:** FIIUH enrolled older adults distribution by type of SUNFRAIL alerts.

SUNFRAIL tool alerts	*n* (%)
Polypharmacy	54 (100)
Weight loss	4 (7.4)
Reduction in physical activity	22 (40.7)
Adherence to medical visits	14 (25.9)
Falls	8 (14.8)
Memory loss	24 (44.4)
Loneliness	16 (29.6)
Support network	11 (20.3)
Socio-economic conditions	10 (18.5)

### 3.2 Persona profile

**Figure 1:**
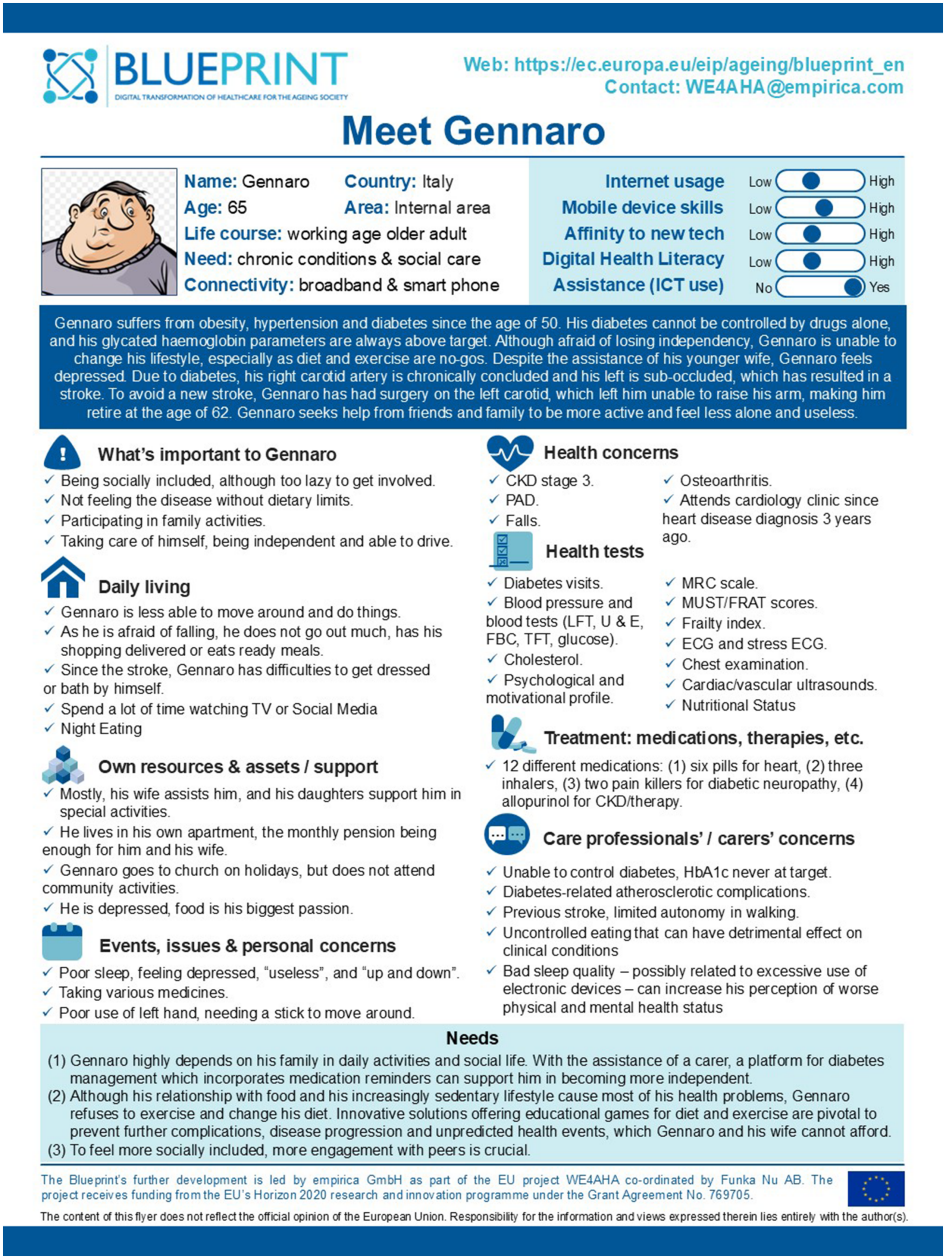
Gennaro’s persona profile card.

Using the Blueprint method and drawing on the SUNFRAIL+ questionnaire results of the sub-cohort, the Focus Group (FG) constructed a fictitious but realistic profile, named ‘Gennaro’. This persona was inspired by common characteristics and unmet needs observed in patients with multimorbidity and polypharmacy, and was iteratively refined through multidisciplinary discussion. Gennaro is a 78-year-old man with obesity, hypertension, and poorly controlled type 2 diabetes, complicated by cardiovascular disease and a previous stroke, which reduced his autonomy and forced early retirement. Despite the support of his wife, he reports depressive symptoms and feelings of loneliness. Living in a suburban area, in a third-floor apartment without a lift and with poor transport connections, he struggles to access healthcare services and maintain social contacts. This persona highlights unmet needs in pharmacological management, physical activity, sociability, disease self-management, and psychological well-being. These needs were systematically linked to potential mHealth and digital solutions identified in the subsequent analysis (Figure 1).

### 3.3 Applicable digital health solution

Based on the persona profile and SUNFRAIL+ data, the FG systematically mapped digital health solutions to the identified unmet needs. The following categories summarize the main solutions, which were organized by domain and technology to ensure a clear link between needs and interventions.

#### 3.3.1 Shared care plan

The Shared Care Plan (SCP) was identified as a priority to address fragmented communication between professionals and patients, allowing shared goal-setting, monitoring, and integration of data from medical devices.

#### 3.3.2 Telemonitoring

Telemonitoring solutions, including wearables and home devices, were proposed to improve real-time monitoring of clinical parameters and support proactive treatment adjustments.

#### 3.3.3 Medication adherence

Mobile apps and smart pillboxes were highlighted as strategies to simplify medication management and improve adherence in polypharmacy.

#### 3.3.4 Physical activity

Adapted physical activity programs delivered via apps or VR-based rehabilitation tools were suggested to counter sedentary behaviors and support functional recovery.

#### 3.3.5 Healthy lifestyle coaching

Digital coaching platforms were proposed to sustain diet and exercise adherence through personalized feedback and progress tracking.

#### 3.3.6 Promotion of motivation to change in self-care processes

Motivational apps based on self-regulation and interactive tasks were identified as tools to strengthen autonomy and adherence to self-care.

#### 3.3.7 Ambient assisted living (AAL)

Ambient Assisted Living (AAL) systems were identified as relevant for supporting autonomy, safety, and fall prevention among older adults. The FG emphasized their potential to integrate information from daily activities and living environments, providing timely alerts and promoting safer ageing at home and in the community.

A summary of the identified needs and digital solutions is provided in Table 4, which links each domain (clinical, psychological, social, environmental) with potential technological interventions. This analytical structure reduces redundancy and highlights the alignment between unmet needs and feasible solutions.

**Table 4:** Summary table of identified technological solutions.

Use case domains	Identified unmet needs	Current clinical tasks	Functionalities of the supportive digital solutions
Professionals/Carers Concerns	Improving accessibility to healthcare	Gennaro must physically go to the health professional’s outpatient clinic to receive instruction on his diseases	A shared care plan supported by IT will allow the Gennaro to consult the specialist doctors remotely, communicating personal health information and integrating the clinical data collected by him at home into the doctor’s electronic health record. The shared care plan will allow Gennaro to schedule appointments online, request therapy updates, and monitor therapeutic goals.
Health concerns	Improving the ability to diseases self-manage	Gennaro has a low awareness of his health status which leads him to not follow doctors’ prescriptions and to adopt a lifestyle that is not suitable for his health condition.	A telemonitoring system on smart-phone, integrated with medical devices connected in Bluetooth allows the measurement of blood pressure, blood sugar, pulse oximetry, heart rate in a simple way, activating notifications based on the patient’s health condition. The telemonitoring system is connected to the electronic medical record of the professional, who will have access to a lot of information during follow-up visits.
Treatments	Polypharmacy management and adherence	Gennaro often forgets to take his medications. He is currently helped by family members and by a paper diary to manage the medications to be taken.	Mobile apps allow to manage the drugs treatment prescribed by professionals in a simple way. The patients access a tab of the medication, with the possibility of scheduling notices of the shots. Some apps are connected with national drug nomenclatures and offer a barcode reader to access all the information of a medication.
			Smart pillbox features lights that indicate which pills to take and how many. Smart Pillbox can be connected via Bluetooth with the mobile app, allowing the patient to receive notifications and reminders on the phone
Daily Living	Physical activity	Gennaro is a sedentary overweight person who would need to follow a personalized exercise program based on his pathologies and disabilities.	Platform for Adapted physical activity (APA) allow patient to access the exercise programme from home via a web-based interface or mobile app, which contains video tutorials of the exercises. The platform allows exchanges between patients and the physician through a dedicated communication channel.
			The smartwatch monitors the number of steps, the intensity of numerous physical exercises, the period of inactivity, and the main vital parameters. The use of the smartwatch incentivizes patients to physical activity and closer monitoring of their health status. Data can be recorded and transmitted thanks to the Shared Care Plan or Patient Portal
What is important to Gennaro	Adherence to healthy lifestyle	Gennaro has a lifestyle that is not in line with his health condition. He does not follow nutritional and medical prescriptions to prevent exacerbations of the diseases he suffers from.	Mobile apps allow to remind nutritional prescription by professionals in a simple way. The patients access a menu containing tailored nutritional advice. In addition, scheduled notices will remind the adequate time to go asleep and the deadline for daily time to spent on electronic devices.
Personal Concerns	Promoting motivation to change in self-care processes	Gennaro has motivational profile based on ambivalence. On the one hand, he is concerned about his loss of independence and fears the consequences of his deteriorating health. On the other hand, he has a psychological resistance to lifestyle changes such as diet and exercise. This conflict reflects an intrinsic low motivation towards self-management and a sense of impotence in relation to disease management.	Motivational Interviewing-based mobile app with the purpose to promote self-care change motivation, engagement, and therapeutic adherence.
			The user accesses a personalized menu which proposes different personalized tasks which are related to life objectives deemed as significant by the person. This allows for ana active, IT assisted, self-regulation process which encompasses important relational functions and supports the person’s sense of autonomy, competence and relatedness to promote subjective well-being
Own resources and assets	Built environment	Gennaro lives in a neighbourhood that is not suitable for his needs, and his house is inappropriate for his diseases.	Fall detection systems: ambient sensor-based monitoring technologies (e.g., passive infrared motion sensors, video sensors, sound sensors, floor sensors), are designed to detect falls among older adults
			Mobile geolocation app that indicates the safest pedestrian routes tailored to his needs, such as proximity to green areas, sidewalk width, and access to community social places. The app aims to encourage an active lifestyle through safe and green walking paths, while also addressing social integration by promoting community activities that foster a sense of integration and active involvement for the patient.

### 3.4 Digital intervention

The FG identified requirements for a digital intervention to support integration of the proposed solutions. Specifically, participants outlined the types of data, the tools for data collection, the implementation contexts, and the interoperability standards needed for a shared database capable of managing inputs from multiple sources (e.g., clinical settings, self-monitoring devices, and wearables). These elements are summarized in Table 5.

**Table 5:** Digital intervention data set.

Unmet needs	Data	Tools	Settings of the service provision	Interoperability requirements
Improving accessibility to healthcare	Patients’ medical records	Shared care plan	Outpatient clinic and Patient’s home	Professional EHR
Improving the ability to diseases self-manage	Blood pressure (Dia/Sys)	Smart blood pressure monitor	Patient’s home	Patient’s Shared Care Plan and Professional EHR
	Heart rate			
	Blood tests	Smart Glucometer		
	BMI	Smart scale		
Polypharmacy management and adherence	Level of adherence to drug therapy	Smart pill box	Patient’s home	Patient’s Shared Care Plan and Professional EHR
		SAT Scale		
Physical activity	Calories burned	Smart watch	Patient’s home	Patient’s Shared Care Plan and Professional EHR
	Number of steps, distance (km, miles), and Elevation			
	Fitness Level via VO2 Max test			
	Electrocardiogram—aFib detection			
	Heart rate variability			
	SpO2—oxygen saturation level			
	Adherence to physical exercise programme	Platform for Adapted physical activity		
	Tests to assess limb functionality	Short Physical Performance Battery	Outpatient Clinic	Professional EHR
	Hand grip test	Dynamometer		
	Fall Risk	Timed Up and Go Test		Professional EHR and Social Care Record
		Balance Test		
Adherence to healthy lifestyle	Sleep cycles: deep, light, Sleep interruption	Smart watch	Patient’s home	Patient’s Shared Care Plan and Professional EHR
	Heart rate zone and sleep heart rate			
	Sleep apnea			
	Meals number/day (n)	Lifestyle coaching mobile app	Patient’s home	Patient’s Shared Care Plan and Professional EHR
	Meals description (n)			
	Prohibited food			
	Added salt.			
	Coffee intake (cups)			
	Nutritional assessment	Mini Nutritional Assessment Survey	Outpatient clinic	Professional EHR
	Survey on adherence to nutritional prescription	PREDIMED Survey		
Promoting motivation to change in self-care processes	Support in healthcare relationship	Qualitative interview and quantitative survey	Outpatient clinic	Internet connection with protocol for data transmission to Patient portal or Shared therapeutic plan
	Basic Psychological Needs			
	Stage of Change and Motivation to a Healthier Lifestyle			
	Self-Perceptions of Aging			
	Engagement in Healthy Aging			
	Perceived Autonomy			
	Adherence to therapy			
	Depression and anxiety screening			
Built environment	Social Support Network	Social Support Scale	Outpatient clinic	Professional EHR and Social Care Record
	Ability to perform essential daily tasks	Activity of Daily Living Scale		
	Neighbourhood features assessment	Qualitative and Quantitative interviews for data collection	Outpatient clinic and patient’s neighbourhood inspection	Professional EHR and Social Care Record
	Building/Home features assessment	Qualitative and Quantitative interviews for data collection	Outpatient clinic and patient’s building/home inspection	

## 4 Discussion

This study explored the unmet needs of older adults with multimorbidity, linking SUNFRAIL+ data with a FG-based persona development process, and mapped potential digital solutions. Multimorbidity, highly prevalent in older adults (43), is closely associated with polypharmacy and adverse outcomes such as increased mortality, reduced quality of life, and functional decline (44). Our findings suggest that polypharmacy, memory loss, reduced physical activity, and isolation represent prominent challenges in our study population. The high prevalence of polypharmacy and self-reported memory loss in our cohort underscores the interplay between cognitive vulnerability and complex therapeutic regimens. Similarly, reduced physical activity and social isolation reflect multidimensional frailty that can accelerate functional decline. These findings were embodied in the persona ‘Gennaro’, which synthesized the most recurrent trajectories of risk and unmet needs identified through SUNFRAIL+ data and FG discussion. In this context, the “Personas” approach, developed by the “Blueprint on Digital Transformation in Health and Care in an Aging Society,” offers a concise and intuitive method to identify unmet needs among specific subgroups of patients.

### 4.1 Principal findings and persona development

While the persona approach provided a useful synthesis of quantitative and qualitative findings, it remains a theoretical construct. In our study, patient co-design was not directly included, which may limit ecological validity. Future studies should integrate patients’ perspectives to refine and validate personas against real-life experiences. Despite this limitation, the efforts of the interdisciplinary focus group in the present study allowed us to identify the IT solutions and the dataset pivotal to implement a digitally supported intervention addressing the identified needs. This approach aims both to enhance cost-effective healthcare services (45) and facilitate multidisciplinary integration in the management of non-communicable chronic diseases (46).

### 4.2 Digital solutions for clinical management

According to FG insights, shared care plans and telemonitoring systems were identified as potentially supporting patients’ day-to-day self-management and potentially assisting healthcare professionals in treatment optimization, though this requires prospective evaluation. Adherence to multiple medications therapy in multimorbid NCD patients is challenging (47). In our FG, medication adherence emerged as a critical issue for multimorbid patients. Among the solutions identified, smart pillboxes and mobile apps were considered relevant to simplify complex regimens. This aligns with previous evidence showing their role in reducing adverse events and improving compliance (48). The FG also emphasized the importance of supporting clinicians in managing polypharmacy. Clinical decision support systems were identified as potential enablers, in line with prior studies showing their value in improving prescribing and deprescribing practices (49, 50). Diabetes represents one of the prevalent chronic conditions within multimorbidity scenarios (51). To effectively manage diabetes, non-invasive blood glucose monitoring may help empower patients to make informed decisions regarding dietary choices, physical activity levels, and medication usage (52). Maintaining glucose levels within the target range contributes to mitigating additional health complications associated with diabetes (45).

### 4.3 Supporting lifestyle and behavioral change

Proper nutrition is essential for managing chronic diseases and improving overall health. Mobile apps can provide tailored nutritional advice and reminders and may support adherence to dietary prescriptions. These tools facilitate regular monitoring and adjustment of dietary plans based on the patient’s health status and taste preferences (53). Sedentary behavior is linked to an increased risk of multimorbidity and falls among older adults, highlighting the importance of public policies promoting physical activity (54). Adapted physical activity programs, administered at home through a web-based platform interface, coupled with smartwatches or other wearable monitoring devices, may encourage physical exercise and may enhance the overall health status of patients in some contexts, subject to validation (55). Self-care is a dynamic and complex process that requires psychological adjustment of NCD patients to their medical conditions (56). Motivational processes and emotional self-regulation are relevant both to adapt to disease conditions and to manage and maintain self-care behaviors (57). Personal motivation is a key element for patients to be ready for changes in disease self-management. Motivational Interviewing (MI) may constitute a very appropriate intervention in those cases in which the behavioural changes (i.e., dietary changes, physical activity, etc.) are hindered by ambivalence, resistance and denial (58). MI enhance patient engagement and self-care by providing personalized tasks and goals, fostering a sense of autonomy and competence. MI contribute to address psychological barriers to lifestyle changes, such as fear of losing independence or depressive symptoms. Technology-delivered motivational interviewing interventions (TAMIs) offer considerable potential to reduce costs, minimize therapist and training burden, and expand the range of clients that may benefit from adaptations of MI (59).

### 4.4 Environmental and technological integration

Beyond psychological support, the FG also emphasized environmental and technological solutions that directly sustain autonomy in daily life. Our FG highlighted the relevance of Ambient Assisted Living (AAL) systems to promote autonomy and safety in older adults. This finding is consistent with studies on indoor monitoring and data integration (60, 61) and on outdoor navigation support and fall prevention (62, 63). Nonetheless, the integration of heterogeneous data sources remains a challenge, as underlined by El Murabet et al. (64). Last but not least, the built environment significantly influences the health and well-being of older adults, both in outdoor and indoor settings (65, 66). Outdoor activities, such as walking, shopping, socialising, and engaging in physical exercise—are essential to prevent functional decline in older adults. Providing an adequate functional mix within neighbourhoods, together with accessible parks and green spaces, creates opportunities for movement and spatial orientation in open areas, while alternative, comfortable, and safe mobility systems support high levels of walkability. In addition, the smart-home modification process for ageing in place involves not only structural refurbishment or the rearrangement of the housing layout to increase usability and safety, but also the installation of smart technology devices (67, 68). Therefore, qualitative and quantitative assessments of neighbourhood and home features are instrumental in identifying modifications that support ageing in place. Technologies such as fall-detection systems and geolocation apps may help enhance safety and support an active lifestyle by reducing fall and wandering risks, pending prospective validation. Next, steps include multi-site, cross-context studies to co-design and prospectively validate the mapped digital options, evaluating feasibility, usability, acceptability, and contextual fit across different healthcare systems.

### 4.5 Implications for implementation and policy

To move beyond isolated pilots, digitally supported home care should be embedded within governance and reimbursement frameworks; otherwise adoption risks remaining fragmented and time-limited (69). Integration through platforms interoperable with the Electronic Health Record (EHR) would allow clinicians to prescribe digital interventions within existing care pathways and reduce siloed use. Although this study did not assess social prescribing of digital tools, such an approach could be promising for prevention and health promotion; however, organisational and cost evaluations are required before scale-up (70). Future work should also broaden clinical perspectives by involving additional specialties (e.g., vestibular, somatosensory, visual) to capture needs that influence independence and well-being in older adults (71–73). In low-resource contexts, staged adoption can prioritise low-cost, high-impact functions (e.g., medication reminders, basic vitals logging) delivered via SMS/IVR or USSD and offline-first workflows. A minimal dataset (only safety-critical fields) and interoperability through open standards reduce technical burden and vendor lock-in. Task-shifting to community health workers, short micro-trainings for patients/caregivers, and clear governance/reimbursement micro-paths (e.g., bundles or capitated add-ons) can improve feasibility. A lean monitoring plan (simple indicators for uptake, adherence, flags) supports iterative scale-up. As a single-centre Italian study, the present findings are context-dependent. The methodological approach (persona-guided, expert-informed mapping) is portable, but content and priorities will require local adaptation to reflect cultural norms, service organisation, and reimbursement rules.

### 4.6 Limits

This study has several limitations. First, the persona and digital options were developed through expert insight rather than direct patient co-design; co-design and end-user validation are necessary next steps. Second, qualitative data reflect a single-center, professionals-only focus group in Italy, which may limit transferability to other contexts. Transferability may also be constrained by cultural factors in aging and care-seeking, which were not explored in this study. Third, the quantitative component is descriptive and restricted to a polypharmacy subgroup (*n* = 54), and therefore does not support inferential claims. Fourth, although multidisciplinary, the focus-group composition may have under-represented some clinical domains (e.g., vestibular, somatosensory, visual), and priority-setting reflected resource constraints. Fifth, we adopted reflexive thematic analysis and therefore did not compute inter-rater reliability coefficients; rigor was supported via double coding, consensus meetings (with third-investigator adjudication when needed), an audit trail, member-checks, and a search for negative cases. Sixth, the study did not assess social prescribing or the economic/organizational implications (e.g., cost-effectiveness, reimbursement models) of implementation. Despite these safeguards, residual interpretive bias may persist; future work will include patient/caregiver co-design, multi-site validation, and holistic feasibility assessment.

## 5 Conclusion

This study provides a structured methodological basis for identifying unmet needs in older adults with multimorbidity and mapping tailored digital health solutions. By integrating SUNFRAIL+ data with FG insights through the Blueprint persona approach, we described critical challenges such as polypharmacy, memory loss, reduced physical activity, and social isolation, and linked them to concrete technological options including telemonitoring, shared care plans, medication adherence tools, and Ambient Assisted Living systems. These findings suggest that, when embedded in a holistic model of care, digital interventions may help support adherence, continuity of care, and autonomy and well-being, pending co-design and prospective validation. While further validation and patient co-design are required, this approach represents a promising pathway to strengthen the long-term management of multimorbidity.

## Data Availability

The raw data supporting the conclusions of this article will be made available by the authors without undue reservation.
