# Supplementary material for: Digital interventions addressing the unmet needs of older adults with multimorbidity: a mixed-methods persona design approach
Source: Front Public Health. 2025 Nov 17;13:1637748. doi: 10.3389/fpubh.2025.1637748 (PMC12665755; doi:10.3389/fpubh.2025.1637748)
Supplement: Supplementary file 1 [file Data_Sheet_1.ZIP › Supplementary Materials_S1_Professionals_FG_Prompts_and_Stimuli.docx]

**Supplementary Material S1
Professionals’ Focus Group discussion guide (verbatim prompts) and stimulus technologies**

**S1.1 Semi-structured discussion guide — Professionals (verbatim prompts)**

**1. Opening & roles.** *Please state your role and experience with older adults with multimorbidity.*

**2. Unmet needs (overview).** *From your professional perspective, which unmet needs (clinical, psychological, social, environmental) are most frequent and impactful?*

**3. Medication & adherence.** *Which medication-related issues (e.g., polypharmacy, reconciliation, reminders) most hinder adherence? How are these issues currently managed?*

**4. Care coordination.** *Where do you observe breakdowns in care coordination and information continuity across primary care, specialists, and community services?*

**5. Daily-life impact.** *How do mobility, home/neighborhood features, and social participation influence self-management and safety?*

**6. Digital literacy & feasibility.** *Which digital literacy barriers (patients/caregivers) and service-delivery constraints limit feasibility at scale?*

**7. Candidate digital functions.** *Considering potential tools, which digital functions (e.g., reminders, tele-follow-up, symptom tracking, shared care plan) best map to the needs identified—and why?*

**8. Data & interoperability.** *What minimum dataset and interoperability are necessary for safe, useful integration into routine care?*

**9. Barriers/facilitators.** *What organizational, technical, economic, or cultural barriers do you anticipate? Which facilitators could mitigate them?*

**10. Acceptability & prioritization.** *Which options should we pilot first and on what criteria (impact, feasibility, equity)?*

**11. Closing — member-check.** *Do these takeaways fairly reflect our discussion? Is anything missing or misrepresented?*

**S1.2 Stimulus technologies shown to professionals**

The following off-the-shelf or near-market technologies were presented as stimuli during the discussion.

| Technology | Intended purpose | Example functions |
| --- | --- | --- |
| Smart pillbox | Medication organization & adherence | Scheduled dose reminders; missed-dose alerts; refill prompts |
| Smartband | Activity & vitals monitoring | Step count; activity minutes; sleep duration |
| Smart scale | Home weight monitoring | Weight trend; simple BMI estimate |
| Wireless blood-pressure monitor | BP self-measurement at home | Guided measurement; automatic logging |
| Pedal trainer (mini cycle) | Low-impact exercise at home | Session duration; cadence |
| SmartTag hub (BLE/NFC) | Home device connectivity | Device pairing; data sync aggregation |
| Medication-management apps | Digital support for medication lists and reminders | Custom schedules; drug interaction alerts (if available) |

**Notes**: No proprietary endorsements are implied. Screenshots can be added if required by the journal; ensure removal of any identifying information.

**S1.3 Session structure & timing (professionals’ focus group)**

Approximate duration: 90 minutes. Structure: agenda and introductions → elicitation using prompts and stimulus technologies → consolidation of key points → member-check of takeaways.
