# Supplementary material for: Digital interventions addressing the unmet needs of older adults with multimorbidity: a mixed-methods persona design approach
Source: Front Public Health. 2025 Nov 17;13:1637748. doi: 10.3389/fpubh.2025.1637748 (PMC12665755; doi:10.3389/fpubh.2025.1637748)
Supplement: Supplementary file 1 [file Data_Sheet_1.ZIP › Supplementary Materials_S2_Qualitative Codebook and Theme Map.docx]

**Supplementary Material S3**

**Qualitative codebook and theme map**

**Compliance note.** In line with standard practices for preserving participant anonymity in qualitative research, this appendix provides the analytical codebook and theme map without verbatim excerpts. The full set of authenticated, anonymized extracts is available to the editors and reviewers upon reasonable request

**S3.1 Codebook (codes → definitions → linked theme)**

| Code | Definition | Theme |
| --- | --- | --- |
| Polypharmacy reconciliation | Issues reconciling medications, duplications, or changes across care settings. | Medication complexity & adherence |
| Missed-dose triggers | Events and patterns leading to missed or delayed doses at home. | Medication complexity & adherence |
| Shared care plan gap | Lack of an accessible, up-to-date care plan shared across providers and services. | Care coordination & information continuity |
| Transitions handover | Breakdowns during transitions (hospital → community; specialist → GP). | Care coordination & information continuity |
| Device/app literacy barrier | Limited ability of patients/caregivers to use devices or apps effectively. | Digital literacy & access |
| Connectivity/access barrier | Connectivity, device availability, or cost constraints limiting digital access. | Digital literacy & access |
| Activity pacing & fatigue | Balancing activity, rest, and symptom fluctuations in daily life. | Self-management & daily functioning |
| Home safety & environment | Environmental factors (layout, lighting, hazards) affecting independence and safety. | Self-management & daily functioning |
| Minimum dataset (MDS) | Core health/behavioral data needed for safe, useful monitoring and handovers. | Data & interoperability requirements |
| Interoperability pathway | Standards and workflows to exchange data with EHR/primary care systems. | Data & interoperability requirements |
| Workforce burden | Staff time/effort required to deploy and sustain digital solutions. | Implementation feasibility |
| Cost & reimbursement | Economic feasibility and reimbursement models needed for at-home digital care. | Implementation feasibility |
| Privacy/consent comfort | Acceptability related to data sharing, privacy, and consent clarity. | Acceptability & prioritization |
| Pilot selection criteria | Criteria to prioritize which digital options to test first. | Acceptability & prioritization |

**S3.2 Theme map (textual)**

Central topic: Unmet needs in older adults with multimorbidity → Persona-guided digital options.
Themes and relations:

1) Medication complexity & adherence ↔ 2) Care coordination & information continuity
 ↘ informs 5) Data & interoperability requirements (MDS, EHR exchange)

3) Digital literacy & access → moderates adoption across all functions

4) Self-management & daily functioning ↔ 1) Medication adherence (routines, cues)

6) Implementation feasibility (workforce, cost/reimbursement) → constrains scalability

7) Acceptability & prioritization → guides pilot selection and sequencing

**Note**: Authenticated anonymized extracts can be provided to the editors upon reasonable request.
